# Analysis of influencing factors of clinical pregnancy rate in second cycle intrauterine insemination

**DOI:** 10.1097/MD.0000000000040406

**Published:** 2024-11-08

**Authors:** Lan Li, Min Wang, Haiyun Wang, Min Yong, Wenping Wang

**Affiliations:** a Department of Obstetrics and Gynecology, Affiliated Hospital of North Sichuan Medical College, Nanchong, Sichuan, China.

**Keywords:** clinical pregnancy rate, influencing factors, intrauterine insemination

## Abstract

To explore the factors influencing the clinical pregnancy rate in the Reproductive Center of the Affiliated Hospital of North Sichuan Medical College in the second cycle, a retrospective analysis of the clinical data of 175 patients who underwent the second cycle of intrauterine insemination (175 cycles) from July 2019 to July 2022 was performed. According to whether the patients reached clinical pregnancy, they were divided into the pregnant group (32 cycles) and non-pregnant group (143 cycles). The age, infertility years, infertility factors, infertility type, number of sinus follicles, intimal thickness, intimal type, basal follicle-stimulating hormone, basal luteinizing hormone, basal estradiol, stimulation regimen, season, body mass index, and male semen quality were statistically analyzed between the 2 groups. The number of intrauterine insemination (IUI) in the second cycle was 175, and the clinical pregnancy rate was 18.3% (32/175). The age of the pregnant group was lower than that of the non-pregnant group [(26.7 ± 1.07) vs (30.56 ± 0.51), *P* = .05]. The clinical pregnancy rate in patients aged ≤30 years was significantly higher than that in patients aged > 30 years [28.6% vs 7.1%, *P* < .05]. Among infertility factors, the pregnancy rate of patients who underwent IUI due to female factors was significantly higher than that of male factors, combined factors (both male and female), and unexplained infertility, and the differences were statistically significant [37.1% vs 22.4%, 18.2%, 3.2%, *P* < .05]. In multivariate logistic regression analysis, age and infertility factors were independent influencing factors of clinical pregnancy rate in the second cycle of IUI. In, while in artificial insemination, female age and infertility factors were important factors affecting the clinical pregnancy rate of the second cycle of IUI. If the outcome of the first cycle is not pregnant, the female age is <30 years, infertility is the female factor, and the second cycle of IUI can be considered.

## 1. Introduction

Infertility is defined as failure to achieve a clinical pregnancy for more than 1 year without unprotected sexual intercourse. In recent years, the incidence of infertility has been increasing owing to the influence of environmental factors.^[1]^ Studies show that infertility or subfertility affects 186 million couples worldwide,^[1]^ accounting for one-quarter of couples of childbearing age.^[2]^ Intrauterine insemination (IUI) involves washing semen and removing dead sperm, white blood cells, and seminal plasma, which are then transported the treated semen into the uterine cavity through a catheter. There are various clinical indications for IUI, including male factor infertility, endometriosis, unexplained infertility, and unilateral tubal defect, etc.^[3]^ Intrauterine artificial insemination is widely used worldwide because of its simplicity, low invasiveness, low cost, and low morbidity rate. However, the pregnancy rate varies according to the indications, ovarian stimulation, and semen parameters.^[4]^ Studies have indicated that the success rate of IUI is lower than that of in vitro fertilization (IVF).^[5]^ After the failure of the first cycle of IUI, couples who do not become pregnant after the next cycles of IUI finally choose IVF. Too many cycles of IUI not only increases the financial burden of patients but also delays the timing of IVF. Therefore, the next step for couples who are not pregnant in the first cycle of IUI is to perform the second cycle of IUI or directly choose IVF, which is not only the doctors need to consider but also the patient’s concern. However, there is still no research on the factors that influence the success rate of IUI in the second cycle. The main objective of this study was to explore the variables that could influence the success rates of IUI treatment in the second cycle to help doctors to give patients who do not get pregnant after the first cycle of IUI the next treatment plan.

## 2. Data and methods

### 2.1. Clinical data

This retrospective analysis of 175 IUI cycles performed between July 2019 and July 2022 on 175 couples who underwent the second cycle of IUI at the Affiliated Hospital of North Sichuan Medical College. All study couples had at least a 1-year history of infertility. They were candidates for IUI because of mild-to-moderate male semen abnormalities, stage I–II endometriosis, and unexplained infertility. A basic infertility workup included a detailed medical history, physical examination, transvaginal ultrasonography, hormone study, hysterosalpingogram, and semen analysis. Hormonal studies, including follicle-stimulating hormone (FSH), luteinizing hormone (LH), and estradiol (E2), were performed on the second day of the menstrual cycle. At least 1 fallopian tube was patented using uteroviduct ultrasound or liodography. All male patients underwent at least 2 semen tests.

### 2.2. Methods

Controlled ovarian stimulation was performed either with letrozole, gonadotropin alone, letrozole combined with gonadotropin, or without any stimulation in a natural cycle, according to the age of the female patient, menstrual cycle, ovarian function, and previous ovulation induction. Stimulation with letrozole was performed from days 5 to 9. Pure human menopausal gonadotropin initial dose of pure human menopausal gonadotropin started daily from the second or third day of menstruation and was adjusted in a timely manner according to follicular response. Once a follicle ≥18 mm in size was identified, a serum LH test was performed: if the LH level was <20 IU/mL, human chorionic gonadotrophin (HCG) 8000–10,000 IU or recombinant HCG 250 µg was given as an ovulation trigger and a single IUI was planned 36 hours later. If the LH level was ≥20 IU/mL, HCG trigger was not administered, and IUI was performed the next day.

After 2–7 days of abstinence, semen was collected in the laboratory. The semen was centrifuged at 37 °C, 300–500/rpm, for 5–10 minutes. The upper layer was then discarded. The lower layer was resuspended in 2 mL medium and further centrifuged for 2 times. The upper layer was discarded in each case. Finally, the precipitates were replaced in 0.5–1 mL medium and placed in a humidified incubator at 37 °C for 30–60 minutes to allow the sperm to swim upward. The washed sperm was then placed in the IUI catheter.

With the patient in the lithotomy position, a speculum was introduced into the vagina after routine vulvar disinfection. The vagina was flushed with saline, and the cervix was cleaned with a dry cotton ball. A soft catheter was used for IUI insemination. The proximal end of the catheter was introduced into the uterine cavity and the prepared semen sample (0.5 mL) was slowly injected over approximately 2 minutes. Ultrasound B was performed on the second day after IUI surgery to determine ovulation. After insemination, each patient received 20 mg of oral ditregesterone daily. Plasma β-HCG levels were measured routinely. If the result was positive, the assay was repeated 3 days later to check for an increase in the HCG level. Clinical pregnancy was defined as the presence of intrauterine gestational pregnancy confirmed by ultrasound. This study was approved by the Reproductive Medicine Ethics Committee of the Affiliated Hospital of North Sichuan Medical College.

All statistical tests were performed using the SPSS 24 software. For continuous data, the Kolmogorov–Smirnov normality test was first employed to ascertain the distribution. Continuous data were expressed as mean ± standard deviation or median (25th–75th percentiles). Normally distributed continuous data were analyzed using the *t* test, and abnormally distributed continuous data were analyzed using the Mann–Whitney *U* test. Categorical data are presented as numbers or percentages. Categorical data were analyzed using Pearson chi-squared or Fisher exact test. Multivariate logistic regression analysis and adjusted odds ratios were used to define independent predictive factors of the outcome variables. The 95% confidence intervals (CIs) were calculated. Statistical significance was set at *P* < .05.

## 4. Results

We analyzed 175 IUI-H cycles in 175 couples during the study period, representing a pregnancy rate of 18.3%. The mean female age was 29.7 ± 1.07 years (range, 22–38 years). The mean duration of infertility was 2.3 ± 1.3 years (range, 1–9 years). The age of women in the pregnant group was lower than that in the non-pregnant group, and the difference was statistically significant [(26.7 ± 1.07) vs (30.56 ± 0.51), *P* < .05]. The pregnancy rate (PR) for the female aged ≤ 30 years was 28.6% (*P* < 0.05). The main reasons for infertility were male sex (22.4%), female sex (37.1%), unexplained (3.2%), and combined factors (both male and female) (18.2%). Etiology is a powerful predictor of a successful pregnancy. Couples with female etiology had a higher pregnancy rate than others (37.1%; *P* < .05). The lowest pregnancy rate was obtained for unexplained infertility (3.2%; *P* < .05) [Table 1]. Female infertility was further classified as ovulatory dysfunction (14.8%) and endometriosis (4.9%). The couples treated for anovulation had the highest pregnancy rate (41.6%; *P* < .05). There were no significant differences in infertility years, sinus follicle number, basal FSH, basal LH, basal E2, basal intimal thickness, body mass index, or infertility type (*P* > .05) [Table 1].

**Table 1:** Factors affecting pregnancy rates in intrauterine insemination.

Variables	IUI outcome		t/χ^2^	*P*
	Positive (32)	Negative (143)		
Female age (years)	26.7 ± 1.07	30.56 ± 0.51	2.46	.017
Female age (years)				
≤30	26 (28.6%)	65		
>30	6 (7.1%)	78	6.418	.04
Duration of infertility (years)				
<2	10	56		
≥2	22	87	1.245	.26
Infertility etiology				
Unexplained	2 (3.2%)	60		
Male factors	15 (22.4%)	52		
Combined (both male and female)	2 (18.2%)	9		
Female factors	13 (37.1%)	22	18.49	.000
Number of basal follicles	16.12 ± 6.08	17.27 ± 5.33	-0.673	.503
Basal FSH	6.16 ± 0.26	5.36 ± 0.46	1.39	.169
Basal LH	4.73 ± 0.34	5.49 ± 0.67	-0.99	.323
E2 of M2	62.47 ± 5.8	57.53 ± 4.89	0.409	.684
Endometrial thickness of M2 (mm)	4.84 ± 0.2	4.53 ± 0.29	0.647	.52
BMI	22.97 ± 0.8	21.71 ± 0.36	-1.49	.14
Infertility type				
Primary	24 (22%)	85		
Secondary	8 (12.1%)	58	1.049	.306

FSH = follicle-stimulating hormone, E2 = estradiol, LH = luteinizing hormone, BMI = body max index, M = menstruation.

Table 2 shows the results of IUI with male factors. The mean male age was 31.6 ± 3.6 years (range, 26–45 years). The male age of pregnant group was lower than that of non-pregnant group, but the difference was not statistically significant [(30 ± 0.79) vs (32 ± 0.45), *P* > .05]. The total motile sperm count after treatment in the pregnant group was higher than that in the non-pregnant group, but the difference was not statistically significant [(31.29 ± 4.04) vs (26.83 ± 1.72), *P* > .05]. There were no significant differences in abstinence days, male body mass index (BMI), semen volume before treatment, semen concentration before and after treatment, PR before and after treatment, total motile sperm count before treatment, or deformity rate (*P* > .05) [Table 2].

**Table 2:** Factors affecting pregnancy rates in intrauterine insemination.

Variables	IUI outcome		t/χ^2^	*P*
	Positive (32)	Negative (143)		
Male age	30 ± 0.79	32 ± 0.45	1.987	.05
BMI	25.36 ± 0.81	24.64 ± 0.38	-0.821	.414
Days of abstinence				
<3	9 (16.7%)	43		
≥3	23 (18.7%)	100	0.078	.828
Semen volume before treatment	3.33 ± 0.37	2.82 ± 0.15	-1.403	.165
Sperm concentration before treatment (M/mL)	67.27 ± 7.19	64.94 ± 4.93	-0.213	.832
PR before treatment (%)	30.28 ± 4.68	24.57 ± 2.73	-0.927	0.357
Total motile fraction before treatment (million/mL)	74.42 ± 11.64	161.27 ± 92.99	0.443	0.659
Sperm concentration after treatment (M/mL)	59.53 ± 8.15	61.77 ± 3.85	0.25	0.803
PR after treatment (%)	0.898 ± 0.03	0.896 ± 0.03	-0.38	0.7
Total motile fraction after treatment (million/mL)	31.29 ± 4.04	26.83 ± 1.72	-1.09	0.276
Morphology (%)	4.95 ± 0.66	4.42 ± 0.21	-0.99	0.325

BMI = body max index, PR = ratio of progressive motile sperm.

There were no significant differences in the following: endometrial thickness, endometrial type, stimulation regimen, season, and trigger scheme (*P* > .05) [Table 3].

**Table 3:** Factors affecting pregnancy rates in intrauterine insemination.

Variables	IUI outcome Positive (32)	Negative (143)	t/χ^2^	*P*
Endometrial thickness				
<8	2 (9.5%)	19		
8–10	17 (18.2%)	76		
>10	13 (21.3%)	48	1.453	.484
Endometrium type				
A	0	6		
AB	11 (33.3%)	22		
B	9 (18%)	41		
BC	8 (11.5%)	61		
C	4 (23.5%)	13	8.727	.068
Stimulation regimen				
LE	19 (19.2%)	80		
HMG	1 (8.3%)	11		
LE + HMG	6 (35.3%)	11		
Cycle of nature	6 (12.8%)	41	5.1	.165
Season				
Spring	10 (21.2%)	37		
Summer	13 (25%)	39		
Autumn	5 (10%)	45		
Winter	4 (15.4%)	22	4.294	.231
Trigger				
HCG	19 (18.8%)	82		
rHCG	4 (26.7%)	11		
Spontaneous LH peak	9 (15.3%)	50	1.087	.581

LE = letrozole, LH = luteinizing hormone, HMG = human menopausal gonadotropin, HCG = human chorionic gonadotropin, rHCG = recombinant human chorionic gonadotropin.

Multivariate logistic regression analysis revealed that female age and sex were independent determinants of the likelihood of clinical pregnancy. There was a gradual decrease in the pregnancy rate with increasing female age (*P* < .05; odds ratios [OR]: 0.721; 95% CI: 0.559–0.93). Women with female factors were 1.031 times more likely to become pregnant (OR: 1.031; 95% CI: 1.003–1.354) [Table 4].

**Table 4:** Factors associated with the probability of pregnancy (multivariate analysis).

	*P*	OR	95% CI
Female factors	.005	1.031	1.003–1.354
Female age	.012	0.721	0.559–0.93

Statistical significance was set at *P* < .05.

CI = confidence interval, OR = odds ratio.

## 5. Discussion

According to the findings of the present study, 32 clinical pregnancies were achieved after 175 IUI cycles of the second cycle, for a total pregnancy rate of 18.3%. Arzu Yavuz et al^[4]^ reported a clinical pregnancy rate of 4.7%. Merviel P et al^[6]^ reported a clinical pregnancy rate of 14.7%. This study identified the factors influencing pregnancy after intrauterine insemination in the second cycle.

In the present analysis, female age and infertility factors significantly affected the PR of IUI in the second cycle. A PR of 28.6% per couple was observed in women aged ≤30 years and 7.1% in women >30 years of age. The differences were statistically significant (*P* < .05). Wadhwa et al^[7]^ reported that the PR of ≤25 years was 18.9%, while that of ≥35 years was 9.0%. However, some studies have pointed out that the woman’s age did not affect the PR, provided that the woman’s age was <40 years, achieving a PR of 13.7% per cycle and a rate of 4.1% thereafter.^[8,9]^ Multivariate logistic regression in this study showed that female age was an independent risk factor affecting the PR of IUI, and PR decreased with increasing age (OR: 0.721; 95% CI: 0.559–0.93), in agreement with the literature.^[10]^ This may be because the number and quality of follicles decrease with age. Therefore, younger female women, especially those <30 years old, should consider the second cycle of IUI after the failure of the first cycle. However, this study showed that there was no significant correlation between male age and the IUI success rate.

In our series, infertility etiology was shown to be another positive predictive factor for pregnancy. In our study, the PR for female factors was significantly higher than that for unexplained infertility (37.1% vs 3.2%, *P* < .05). In contrast to our results, Kamath^[11]^ pointed out that PR was higher in unexplained and anovulatory infertility patients than in patients with endometriosis and male factors, although the difference did not reach statistical significance. Vlahos et al^[12]^ pointed out that the PR of IUI in anovulation infertility was 19.1%, whereas that in endometriosis infertility was only 9.1%. Ashraf et al^[13]^ pointed out that the PR for patients with anovulation due to polycystic ovary syndrome was higher than that for other factors. In this study, female factor had the highest PR (37.5%), which was divided into anovulation (75%) and endometriosis (25%). The PR of anovulation was higher than that of endometriosis (41.6% vs 25%, *P* > .05), although the difference was not statistically significant. It is clear that controlled ovarian hyperstimulation corrects ovulation, and the endometrial thickness of patients with anovulation is usually within the normal range, resulting in a high IUI success rate.^[11]^ Ashraf^[13]^ also reported that the cumulative pregnancy rate of anovulation was significantly higher than the cycle pregnancy rate. Therefore, according to the appropriate female age and length of infertility, ovulation disorder infertility with at least 1 fallopian tube patency, the second cycle of IUI after the failure of the first-cycle IUI. Although unexplained infertility is a clear indication for IUI, it had the lowest PR in this study. Similarly, some studies have pointed out that IUI for unexplained infertility does not only seem to increase the pregnancy rate, but also increases the economic burden of patients.^[14]^ The UK National Institute for Health and Care Research suggests that couples can directly consider IVF instead of IUI after failing to become pregnant with unprotected sex for 2 years.^[15]^ This study suggests that patients with unexplained infertility can directly consider IVF rather than IUI after failure of the first IUI cycle.

Some studies suggest that the duration of infertility is related to the success rate of IUI. The success rate decreased with an increase in the duration of infertility.^[11]^ However, it is fair to determine this correlation. Stephen EH^[16]^ indicated that BMI has an impact on IUI success. The success rate of BMI > 25 was higher than that of BMI < 25. This is because most obese patients have ovulatory dysfunction, whose PR is higher than that of others.^[17]^ We also found that the BMI of the pregnant group was higher than that of the non-pregnant group, although the difference did not reach statistical significance (22.97 ± 0.8 vs 21.71 ± 0.36, *P* > .05). This study showed that there was no significant difference in FSH levels between the pregnant and non-pregnant groups; however, previous studies have shown that the FSH value reflects the number and quality of female follicles. The higher the value, the lower the probability of pregnancy.^[18]^

Earlier studies have indicated that pregnancy rates were lower in patients with premature LH surges.^[19]^ In this study, we found a PR of 20.3% in ovulation-triggering cycles versus 14.8% in spontaneous LH rise cycles, although the difference was not statistically significant. This is because the determination of ovulation timing is less precise with an LH rise than in ovulation, and triggering high LH levels could correspond to the upward or downward part of the rise.^[19]^ A significantly higher PR after the use of gonadotropins than after clomiphene was found in previous studies (OR: 1.8; 95% CI: 1.2–2.7).^[20]^ Some studies have also indicated that the clinical pregnancy rate of letrozole stimulation is greater than that of clomiphene stimulation.^[21]^ We suggest that the success rate of letrozole combined with gonadotropins was higher than that of letrozole or gonadotropins alone, but the difference was not statistically significant (*P* < .05). Esmailzadeh et al^[22]^ pointed out that during IUI, the mean endometrial thickness on the day of ovulation triggering was significantly higher when pregnancy was achieved (10.1 mm vs 7.7 mm, *P* < .05). This study showed that the PR value increased as endometrial thickness increased during ovulation, although the difference was not statistically significant (*P* > .05).

As for male factors, this study did not find any correlation between male age, male body mass index (BMI), duration of abstinence, and successful IUI pregnancy. Kamath et al^[11]^ pointed out that the total motile count (TMC) after treatment was an independent factor affecting the success rate of IUI, and proposed that most IUI successes occurred when the range of total motile count was 10 × 10^6^/mL to 20 × 10^6^/mL, while PR was the lowest when TMC was <5 × 10^6^/mL. In this study, TMC after treatment in IUI pregnancy group was indeed higher than that in non-pregnancy group, but the difference was not statistically significant (31.29 ± 4.04 vs 26.83 ± 1.72, *P* > .05).

## 6. Conclusions

This study is the first to investigate the factors that influence the clinical pregnancy rate of IUI only in the second cycle. Among them, the IUI clinical pregnancy rate is high, especially in women aged <30 years. The PR of IUI in the second cycle was the highest for infertility factors, especially those with ovulatory dysfunction. The PR of IUI in the second cycle was the lowest in those with unexplained infertility. Therefore, we suggest that for women who are <30 years of age and whose infertility factor is ovulatory dysfunction, the second cycle of IUI can be considered after the failure of the first cycle. If the cause of infertility is unknown, IVF can be considered directly after the failure of the first IUI cycle. However, there may be some errors owing to the small sample size. Therefore, the factors affecting the success rate of IUI in the second cycle require further investigation.

## Author contributions

**Conceptualization:** Lan Li.

**Data curation:** Lan Li, Min Yong.

**Formal analysis:** Lan Li, Min Wang, Min Yong.

**Investigation:** Haiyun Wang.

**Methodology:** Min Wang.

**Supervision:** Min Yong, Wenping Wang.

**Validation:** Min Yong, Wenping Wang.

**Visualization:** Wenping Wang.

**Writing—original draft:** Lan Li.

**Writing—review & editing:** Lan Li.
